# Acute effects of energy drink consumption on microvascular reactivity in young male volunteers at rest: a randomized trial

**DOI:** 10.1590/1414-431X2024e13624

**Published:** 2024-08-23

**Authors:** L. Skaf-Gonçalves, D. Peçanha, D. Kasal, E. Tibirica

**Affiliations:** 1 Instituto National de Cardiologia, Rio de Janeiro, RJ, Brasil; 2 Departamento de Clínica Médica, Universidade do Estado do Rio de Janeiro, Rio de Janeiro, RJ, Brasil

**Keywords:** Endothelial function, Energy drinks, Laser speckle, Microvascular reactivity, Randomized trial

## Abstract

Energy drinks are nonalcoholic beverages whose main ingredients are sugar, taurine, and caffeine. The consumption of energy drinks is increasing worldwide, but only a few conflicting studies have investigated the vascular effects of energy drinks in young adults. The aim of this study was to evaluate microvascular reactivity before and after energy drinks consumption in young healthy male volunteers. This was a cross-sectional prospective study. Microvascular reactivity signals were evaluated in the skin of the forearm using laser speckle contrast imaging with acetylcholine (ACh) iontophoresis before and 90 and 180 min after the randomized consumption of one ED or the same volume of water (control), followed by a postocclusive reactive hyperemia (PORH) test. Thirty-two volunteers were evaluated (age: 25.4±4.3 years). Energy drink consumption prevented the rest-induced reduction in cutaneous vascular conductance over time that was observed in the control group. In the control group, there were significant reductions in microvascular vasodilation at 90 and 180 min compared to baseline (P=0.004), but this was not the case in the energy drink group (P=0.76). Our results demonstrated that the reduction in microvascular conductance associated with prolonged immobility can be prevented by the consumption of one energy drink, highlighting the vasodilator effects of this beverage in young individuals at rest. The between-study variability in terms of the brand of energy drinks and the ingested volume, as well as the method of vascular evaluation and the inclusion criteria, may explain the discrepancies among previous studies on the vascular effects of energy drinks.

## Introduction

Energy drinks are nonalcoholic beverages, and their main ingredients are sugar, taurine, and caffeine (1). These products are marketed as boosters of mental and physical performance and are increasingly used in Western societies, especially by young adults (2). Case reports of adverse effects related to ED consumption affecting the cardiovascular system, including arrhythmia (3), coronary artery vasospasm (4), and myocardial infarction (5), have been described in otherwise healthy young individuals.

The data support the hypothesis that endothelial dysfunction plays a role in the cardiovascular effects of energy drinks consumption and have prompted studies, the results of which are conflicting, to evaluate the actions of these drinks on circulation (6-9). The lack of an adequate control group, the recruitment of individuals with cardiovascular or metabolic diseases, and the use of different methods for evaluating endothelial dysfunction could be factors leading to these discrepancies.

A noninvasive study approach based on skin microcirculation may be appropriate in this setting. Skin microcirculation has been proposed as the earliest segment of the vascular system affected by endothelial dysfunction (10), with a pattern that mirrors systemic microcirculation, including coronary vessels (11).

The present study aimed to evaluate microvascular reactivity before and after the consumption of one energy drink using a technique specifically designed for continuous imaging of the microcirculation (laser speckle contrast imaging [LSCI]). We used water as the control drink (instead of other energy drinks or stimulant beverages) to compare the effects of energy drink consumption with those of water (i.e. an inert liquid) consumption. In addition, we recruited young healthy individuals to account for the inherent effects of resting conditions, which can impose acute detrimental effects on microvascular reactivity (12). Accordingly, we hypothesized that an approach with a homogeneous participant profile, an adequate sample size, and appropriate control and method selection could provide a better understanding of the acute effects of energy drinks on microvascular reactivity.

## Material and Methods

The present study was performed in accordance with the Helsinki Declaration, revised in 2013, and was approved by the Institutional Review Board (IRB) of the National Institute of Cardiology, Brazil, (protocol #CAAE 13135219.0.0000.5272). The study was registered at ClinicalTrials.gov (https://register.clinicaltrials.gov; #NCT05575895). Once deemed eligible to participate in the study, all subjects read and signed an informed consent document approved by the IRB.

The study included thirty-two young, healthy male volunteers recruited between May 2022 and May 2023. A flowchart of the selection of participants is shown in the online supporting material (#NCT05575895). The clinical characteristics of the volunteers are described in Table 1. The inclusion criteria were age between 18 and 35 years and no relevant findings in the medical history. The exclusion criteria were being a competitive athlete, being on regular medication, using illicit drugs, being allergic to coffee, taurine, or any of the components of the selected energy drink, and having a body mass index ≥30 kg/m^2^. Venous blood samples were obtained for biochemical testing in the morning after a 12-h fast.

**Table 1:** Clinical characteristics of healthy volunteers (n=32).

Characteristics	Data
Age (years)	24.9±4.3
Body weight (kg)	76.4±10.2
Height (cm)	176.4±7.8
Body mass index (kg/m^2^)	24.6±2.5
Waist circumference (cm)	84.5±7.1
Eutrophic (n, %)	19 (59)
Overweight (n, %)	13 (41)
Fasting glucose (mg/dL)	89±8
Total cholesterol (mg/dL)	160±30
LDL-cholesterol (mg/dL)	104±27
HDL-cholesterol (mg/dL)	48 (40-57)
Triglycerides (mg/dL)	72 (51-82)
Creatinine (mg/dL)	0.98±0.14
Urea (mg/dL)	29±7
SBP (mmHg)	121±9
DBP (mmHg)	71±9
MBP (mmHg)	88±7
Heart rate (bpm)	63±10

The data are reported as means±SD. Values that did not follow a Gaussian distribution are reported as medians (25th-75th percentiles; Shapiro-Wilk normality test). LDL: low density lipoprotein; HDL: high density lipoprotein; SBP: systolic blood pressure; DBP: diastolic blood pressure; MBP: mean blood pressure.

### Study protocol

The study was carried out in two sessions on separate days, with the ingestion of 250 mL of the energy drink (Red Bull^®^, Brazil) or the control drink (mineral water Minalba^®^, Brazil), defined by simple randomization with an interval of at least two weeks between them. The ingredients of the commercially available energy drink used (250 mL) were 20 g of sugar, 100 mg of taurine, 600 mg of glucuronolactone, 80 mg of caffeine (equivalent to a cup of coffee), and 2 mg of vitamin B6.

Volunteers were asked to fast for 12 h before the examination and not to consume caffeine-containing beverages in the previous 12 h. Participants were also advised not to engage in strenuous exercise 12 h before the examination. The examinations were performed between 8:00 am and 1:00 pm in a quiet room with controlled temperature (23±1°C). The volunteers were then asked to lie down in the supine position for 10 min. Subsequently, blood pressure and heart rate were measured with a validated and calibrated automatic upper-arm blood pressure monitor (Omron M7, Omron Healthcare, The Netherlands). Each patient underwent measurements in the standing (orthostatic) position and three measurements in the sitting position at 1-min intervals. The mean values of the two last measurements were used in the analysis.

Skin microvascular flow and reactivity were assessed before and 90 and 180 min after consumption of the energy drink or control. Before each evaluation, blood pressure and heart rate were measured again.

### Evaluation of microvascular flow and reactivity

The microcirculatory tests were performed in a quiet room at a stable temperature (23±1°C) after a 20-min rest in the supine position. Microvascular reactivity was evaluated using an LSCI system with a laser wavelength of 785 nm (PeriCam PSI system, Perimed, Sweden), and continuous recordings of cutaneous microvascular perfusion changes were measured in arbitrary perfusion units (APUs). The images were analyzed using PIMSoft software (Perimed). A skin site on the ventral surface of the forearm was randomly chosen for the recordings. Hair, broken skin, areas of skin pigmentation, and visible veins were avoided, and two drug-delivery electrodes were installed using adhesive discs (LI 611, Perimed). Acetylcholine (ACh) (2% w/v; Sigma Chemical Co., USA) iontophoresis was performed using a micropharmacology system (PF 751 PeriIont USB Power Supply, Perimed) with increasing anodal currents of 30, 60, 90, 120, 150, and 180 μA, which were administered for 10-s intervals 1 min apart. The total charges for the above currents were 0.3, 0.6, 0.9, 1.2, 1.5, and 1.8 mC, respectively. The dispersive electrode was attached approximately 15 cm from the electrophoresis chamber.

During the postocclusive reactive hyperemia (PORH) test, arterial occlusion was performed with suprasystolic pressure, defined as 50 mmHg above the systolic blood pressure, by applying a sphygmomanometer for 3 min. Following the release of pressure, the maximum flux was measured. PORH tests were always performed after the iontophoresis of ACh to avoid interference with these pharmacological tests, which are limited by local responses in the skin. In contrast, a hyperemic response is a global response that extends to the skin of the entire forearm and could thus interfere with subsequent tests of microvascular reactivity.

Skin blood flow measurements (APUs) were divided by mean blood pressure values to yield cutaneous vascular conductance (CVC), which is herein presented as APUs/mmHg.

### Statistical analysis

The prospective power analysis was based on LSCI data from a previous study by our group (13). This analysis indicated that a sample size of 31 subjects per group (healthy volunteers and patients with dyslipidemia) would have 80% power at the 5% significance level to detect a mean between-group difference of 0.156 APUs/mmHg (standard deviation of 0.219 APUs/mmHg) in cutaneous vascular conductance induced by cutaneous iontophoresis of ACh. The calculations were performed using classical power calculations with the formula $n=f(\alpha ,\beta )\cdot \frac{2s^{2}}{\delta^{2}}$ , where *α* is the significance level, *β* is the power of the test, *f* (*α*, *β*) is a value calculated from *α* and *β* (in this case, 7.9), *δ* is the difference in the means that we should be able to detect, and *s* is the standard deviation found in these previous studies.

The data are reported as means±SD. Variables without a Gaussian distribution, which was determined with the Shapiro-Wilk normality test, are reported as medians (25th-75th percentiles). Statistical analyses of most microvascular parameters, including area under the curve (AUC) and peak and delta values of ACh-induced vasodilation, were performed using two-way ANOVA followed by Tukey's multiple comparisons test, where we considered the interactions of time (before and 90 or 180 min after ingestion) and intervention (control or energy drink). Statistical analysis of blood pressure and heart rate values was performed using one-way ANOVA followed by Tukey's multiple comparisons test. The basal CVC values were analyzed using unpaired *t*-tests. Statistical analyses of the vasodilation curves induced by ACh iontophoresis were performed using two-way ANOVA followed by multiple comparisons (Tukey's multiple comparisons test), where we considered the interactions of time (before and 90 or 180 min after ingestion) for each intervention (control or energy drink). P values <0.05 were considered to indicate statistical significance. The Prism version 7.0 statistical package was used for statistical analyses (GraphPad Software, Inc., USA).

## Results

### Effects of energy drink on cardiovascular parameters

The clinical characteristics of the study participants are described in Table 1. The effects of the acute ingestion of energy drinks on blood pressure and heart rate are described in Table 2. There was a statistically significant interaction effect of treatment over time on systolic blood pressure [F(2, 124)=3.966, P=0.0214] and heart rate [F(2, 124)=14.24, P<0.001)]. In the control group, systolic blood pressure (SBP) was increased at 180 min (P=0.0466) compared to 90 min. The HR in the control group was reduced at 90 min (P=0.0107) and 180 min (P=0.0001) compared to the basal values. In the energy drink group, the HR was lower at 180 min (P=0.0067) than at baseline.

**Table 2:** Effects of acute ingestion of mineral water (Control) or an energy drink on the cardiovascular parameters of healthy volunteers (n=32).

Parameter	Baseline	90 min	180 min
Control			
SBP (mmHg)	121±10	120±8	123±8^&^
DBP (mmHg)	69±8	70±7	70±8
MBP (mmHg)	87±7	86±7	88±7
HR (beats/min)	63±10	60±10*	58±10***
Energy drink			
SBP (mmHg)	121±8	123±7	124±8
DBP (mmHg)	71±8	72±7	71±8
MBP (mmHg)	88±7	89±6	89±7
HR (beats/min)	62±10	60±10	59±10**

The data are reported as means±SD (according to the Shapiro-Wilk normality test). Statistical analysis was performed using two-way ANOVA followed by Tukey's multiple comparisons test. *P<0.05, **P<0.01, ***P<0.001 compared to baseline values. ^&^P<0.05 compared to 90 min. SBP: systolic blood pressure; DBP: diastolic blood pressure; MBP: mean blood pressure; HR: heart rate.

### Effects of energy drink on skin microvascular flow and reactivity

**Figure 1:**
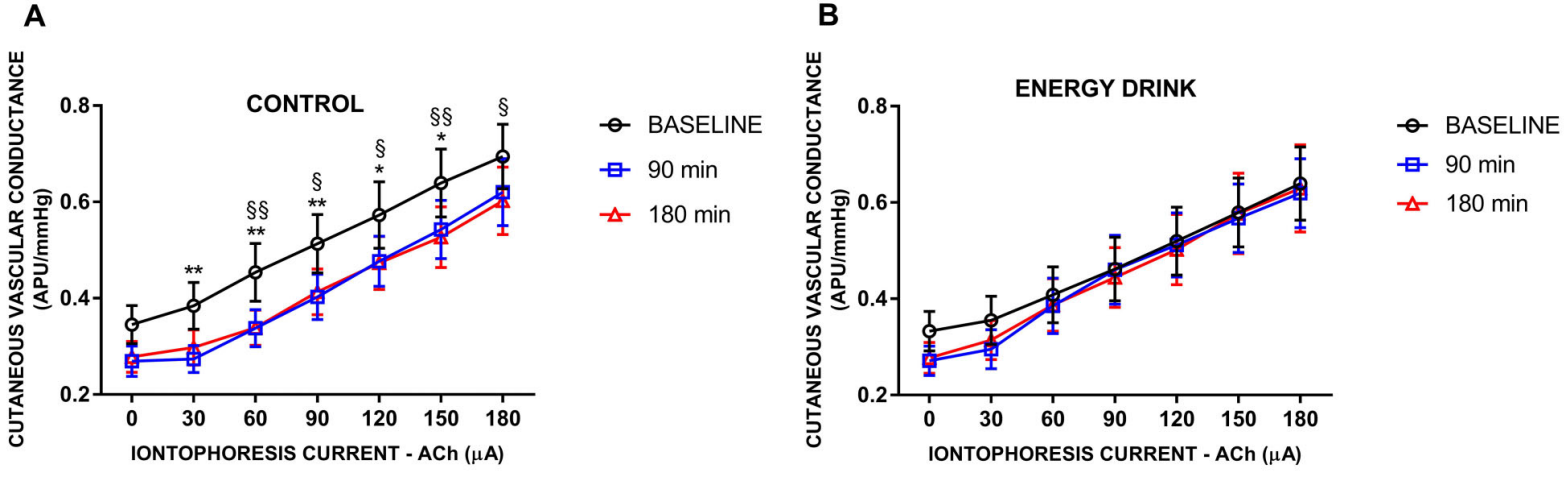
Effects of skin acetylcholine (ACh) iontophoresis on the cutaneous microvascular conductance of healthy volunteers (n=32), reported in arbitrary perfusion units (APUs) divided by the mean blood pressure in mmHg, representing endothelium-dependent microvascular vasodilation before and after acute ingestion of mineral water (control) (**A**) or an energy drink (**B**). The data are reported as medians (95%CI). Statistical analyses were performed using two-way ANOVA followed by Tukey's multiple comparisons test, where we considered the interactions of time (before and 90 and 180 min after ingestion) for each intervention (control or energy drink). *P<0.05, **P<0.01 *vs* 90 min; ^§^P<0.05, ^§§^P<0.01 *vs* 180 min.

Skin iontophoresis of ACh induced current-dependent increases in CVC in both the control and energy drink groups (Figure 1A and B). There was a statistically significant interaction effect between the current intensity and time after ingestion on CVC [F(2, 93)=5.951, P=0.0037] in the control group but not in the energy drink group [F(2, 93)=0.279, P=0.7572].

**Figure 2:**
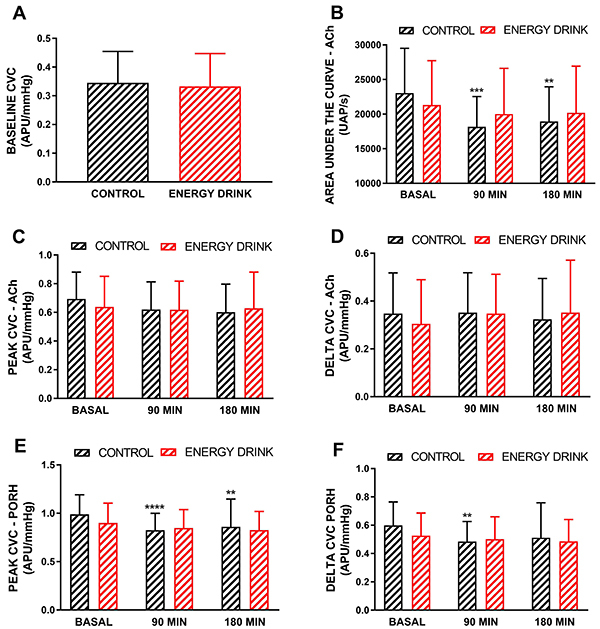
**A**, Baseline cutaneous microvascular conductance (CVC) and effects of acute ingestion of mineral water (control) or an energy drink on the microvascular reactivity of healthy volunteers (n=32). **B**, Area under the curve of microvascular vasodilation induced by skin acetylcholine (ACh) iontophoresis. Peak effects (**C**) and delta effects (peak minus baseline) (**D**) of cutaneous ACh iontophoresis. Peak effects (**E**) and delta effects (peak minus baseline) (**F**) of postocclusive reactive hyperemia (PORH). CVC is expressed as arbitrary perfusion units (APU) divided by the mean blood pressure in mmHg. The data are reported as means±SD (Shapiro-Wilk normality test). Statistical analyses were performed using two-way ANOVA followed by Tukey's multiple comparisons test, where we considered the interactions of time (before and 90 and 180 min after ingestion) and intervention (control or energy drink). **P<0.01; ***P<0.001; ****P<0.0001 *vs* basal values.

The baseline skin microvascular flow values were not different between the control group (0.34±0.1 APUs/mmHg) and the energy drink group (0.33±0.1 APUs/mmHg; P*=*0.62; Figure 2A).

In the control group, the areas under the curve (AUCs) of microvascular vasodilation induced by ACh iontophoresis were 23.043±6.457 APUs/s before the intervention and 18.152±4.390 APUs/s and 18.934±5.005 APUs/s after 90 and 180 min, respectively (Figure 2B). In the energy drink group, the AUC values were 21.327±6.396 APUs/s before the intervention and 20.002±6.599 APUs/s and 20.190±6.727 APUs/s after 90 and 180 min, respectively (Figure 2B). There was a statistically significant interaction effect of treatment over time on the AUC [F(2, 124)=8.343, P=0.0004]. The AUCs in the control group were lower at 90 min (P=0.0001) and 180 min (P=0.0016) than the basal values.

The peak values for microvascular flow induced by ACh iontophoresis in the control group were 0.69±0.19 APUs/mmHg before the intervention and 0.60±0.19 and 0.60±0.19 APUs/mmHg after 90 and 180 min, respectively (P>0.05; Figure 2C). In the energy drink group, the APUs/mmHg values were 0.64±0.21 before the intervention and 0.62±0.19 and 0.63±0.25 APUs/mmHg after 90 and 180 min, respectively (P>0.05; Figure 2C).

The delta values (peak minus baseline) of microvascular flow induced by ACh iontophoresis in the control group were 0.35±0.17 APUs/mmHg before the intervention and 0.35±0.17 and 0.32±0.17 APUs/mmHg after 90 and 180 min, respectively (P>0.05; Figure 2D). In the energy drink group, the values were 0.30±0.18 APUs/mmHg before the intervention and 0.35±0.16 and 0.35±0.21 APUs/mmHg after 90 and 180 min, respectively (P>0.05; Figure 2D).

The peak values of microvascular flow induced by PORH in the control group were 0.99±0.20 APUs/mmHg before the intervention and 0.82±0.17 and 0.86±0.29 APUs/mmHg after 90 and 180 min, respectively (Figure 2E). In the energy drink group, the APUs/mmHg values were 0.90±0.20 before the intervention and 0.85±0.19 and 0.83±019 APUs/mmHg after 90 and 180 min, respectively (Figure 2E). There was a statistically significant interaction effect of treatment over time on the peak values of PORH [F(2, 124)=11.99, P<0.0001]. The peak values of PORH in the control group were lower at 90 min (P=0.0001) and 180 min (P=0.001) than at baseline.

The delta values (peak minus baseline) of microvascular flow induced by PORH in the control group were 0.60±0.16 APUs/mmHg before the intervention and 0.48±0.14 and 0.51±0.24 APUs/mmHg after 90 and 180 min, respectively (Figure 2F). In the energy drink group, the APUs/mmHg values were 0.53±0.16 before the intervention and 0.50±0.16 and 0.49±0.15 APUs/mmHg after 90 and 180 min, respectively (Figure 2F). There was a statistically significant interaction effect of treatment over time on the delta values of PORH [F(2, 120)=4.504, P<0.0130]. Delta values of PORH in the control group were lower at 90 min (P=0.0071) than at baseline.

## Discussion

In the present study of a cohort of young adult healthy male volunteers, energy drinks prevented the MR impairment observed in the control group at 90 and 180 min. Although the acute increase in blood pressure is consistent with the findings of previous studies (7-9), the literature regarding the effects of energy drinks on endothelial function is conflicting. An analysis of the experimental conditions may help to understand the differences among the studies and suggest potential improvements.

A pioneering study by Worthley et al. (7) revealed a decrease in reactive hyperemia index using the peripheral arterial tone (PAT) method one hour after the ingestion of one energy drink. The authors used an energy drink with a similar composition to the beverage used in our study, with the exception that it was sugar-free. Recent data from our group have shown that high carbohydrate ingestion increases microcirculatory vasodilation in healthy subjects, an effect possibly related to insulin stimulus (14). As a consequence, the sugar content in the energy drinks in our study could have counterbalanced potential vasoconstrictive effects. Furthermore, the PAT evaluates systemic pulse waves (15), which do not necessarily represent the microcirculatory milieu evaluated with laser speckle in our study.

Previous results from another group suggested the induction of endothelial dysfunction after one energy drink (9,16,17). Those three studies used one can of a different type of energy drink (Monster Energy^®^), which has twice the volume used in our study and is high in sodium, an ingredient that has been implicated in acute endothelial dysfunction (18). In addition, the lack of water control differed from our approach. Accordingly, the ingestion of fluid may cause autonomic changes related to gastric distention and osmolality, which may affect vascular responses (19).

Another important aspect of most of the previous studies was the use of flow-mediated dilation with ultrasound imaging of the brachial artery. This evaluates the conductance of the macrocirculation, which was previously reported to display a distinct pattern compared to microcirculation analyzed with laser speckle (10). Loss of coherence between the macro- and microcirculation has been described in pathological states, such as sepsis (20,21). An interesting approach would be to simultaneously evaluate both of those vascular territories after energy drink consumption to clarify these apparent inconsistencies.

In the study by Grasser et al. (8), the microcirculation was evaluated via laser Doppler flowmetry two hours after energy drink consumption. The authors found an increased ACh-induced vasodilation when employing the same brand of drink as in our study, but with a greater volume (355 mL *vs* 248 mL in the present study). To the best of our knowledge, the present study is the first to evaluate the vascular effects of an energy drink in microcirculation employing LSCI, a method that combines laser Doppler flowmetry and laser Doppler imaging. This technique is highly reproducible for blood flow imaging (22) and is often preferred because it is easy to use and allows evaluation of a large skin area for fast vascular mapping (23).

All previous studies evaluated a cohort of men and women in a balanced manner. The present study differed in its design, as it evaluated only male subjects. A previous study by our group evaluated young healthy volunteers and showed that female individuals displayed greater vasodilation in response to ACh in the microcirculation than male participants did (24). Indeed, estrogen has been implicated in improved vasodilation through the stimulation of prostacyclin and nitric oxide synthesis, among other mechanisms (25). As a consequence, the acute vascular effects of energy drinks may differ between men and women. Furthermore, the possibility of harmful energy drink effects during pregnancy (26) would also make it ethically difficult to include young women in the study.

One relevant aspect of our findings was the progressive reduction in microvascular conductance over time in the control group, which was not observed after energy drink consumption. Indeed, physical inactivity has been implicated in endothelial dysfunction (27) even in young individuals after intervals as short as 10 min, with microvascular effects extended for up to 1 h after restoring mobility (12). The mechanisms for reduced vasodilation under resting conditions include reduced nitric oxide availability and impaired blood flow, among others (28).

It has been shown that rest-induced vascular dysfunction can be attenuated by previous high-intensity aerobic exercise in young male volunteers (29). Our current results demonstrated that the microvascular conductance reduction associated with immobility can be prevented by the consumption of one energy drink, highlighting the vasodilation effects of this beverage. The clinical significance of these findings and whether they can be translated to other vascular beds, such as the central nervous system, should be further assessed.

### Study limitations

The present study aimed to evaluate the microcirculatory effects of consuming a popular energy drink formulation. In the real world, however, energy drinks are often consumed in quantities far exceeding one can and in combination with alcohol or illicit substances (30,31). These factors can contribute to the adverse effects reported with energy drink consumption and were not evaluated in the present study. Because this study was conducted with male subjects only, the results cannot be generalized to women.

### Conclusion

By using a control group with water and a specific method for microcirculation evaluation, we demonstrated that the ingestion of one can of a popular energy drink can prevent the acute decrease in microvascular conductance induced by immobility. These findings underline the vasodilation effects of this beverage in young healthy individuals. However, further studies are needed to evaluate the vascular effects of energy drinks in the real world, a challenging task considering the differences in compositions among brands, differences in the available volumes, and concomitant consumption of other substances. The simultaneous evaluation of conductance and resistance vessels after the ingestion of an energy drink is warranted to investigate discrepancies among previous studies.
